# Pinyon Engraver Beetle Acoustics: Stridulation Apparatus, Sound Production and Behavioral Response to Vibroacoustic Treatments in Logs

**DOI:** 10.3390/insects12060496

**Published:** 2021-05-26

**Authors:** Ivan Lukic, Carol L. Bedoya, Evan M. Hofstetter, Richard W. Hofstetter

**Affiliations:** 1 School of Forestry, Northern Arizona University, Flagstaff, AZ 86011, USA; ivan.lukic88@gmail.com; 2 School of Biological Sciences, University of Canterbury, Christchurch 8140, New Zealand; c.l.bedoya6@gmail.com; 3 BASIS, High School, Flagstaff, AZ 86001, USA; evanhofstetter@gmail.com

**Keywords:** bark beetle, *Pinus monophyla*, *Ips*, tree protection, mating disruption, pest management

## Abstract

**Simple Summary:**

Acoustic technology is a potential tool to protect wood materials and live trees from colonization by bark beetles and other wood-infesting insects. Bark beetles such as the pinyon engraver beetle *Ips confusus* use chemical and acoustic cues to communicate and to locate potential mates in trees. In this study, we describe the structures and airborne sounds produced by the pinyon engraver beetle, and test the efficacy of vibroacoustic treatments for tree protection against this beetle. Only female beetles possessed sound producing structures, located on the back of the head and inside the thorax. We analyzed and described the airborne sounds, called chirps, produced by females when held by tweezers or placed on their back. We tested a wide variety of vibroacoustic treatments played into logs but these sound treatments did not prevent male entry into logs and did not disrupt female–male interactions, female tunneling behavior, reproduction or egg laying. We suggest further studies if acoustic methods are to be utilized to control this bark beetle.

**Abstract:**

Bark beetles are among the most influential biotic agents in conifer forests, and forest management often focuses on bark beetle chemical communication for tree protection. Although acoustic communication occurs in many bark beetle species, we have yet to utilize acoustic communication for bark beetle control. Here, we describe the stridulatory organs and ‘stress’ chirps of the pinyon engraver, *Ips confusus*, a significant pest and mortality agent of pinyon pine in western North America. Only females possessed stridulatory organs and their stress chirps varied significantly in duration, pulses per chirp, and dominant frequency. We tested an array of acoustic-vibrational treatments into logs but were unable to disrupt male entry into logs or alter female–male interactions, female tunneling, and female oviposition. We found acoustic–vibrational treatments had little effect on *I. confusus* behavior and suggest further studies if acoustic methods are to be utilized for bark beetle control.

## 1. Introduction

Bark beetles (Coleoptera: Curculionidae, Scolytinae) are well-known forest pests due to their ability to kill trees [1,2,3,4]. Bark beetle species that kill trees [5] use aggregation pheromones to coordinate mass attacks on host trees [6,7,8]. Mass aggregation pheromones are released by the pioneer sex which is male (e.g., *Ips* species) or female (e.g., *Dendroctonus* species) depending on the bark beetle genus [9,10]. Interestingly, the non-pioneering sex typically has well-developed stridulatory structures [11,12] and will stridulate (i.e., produce sounds/vibrations) when arriving near or inside the gallery of a pioneer’s entry hole [13,14]. Airborne sounds and stridulatory structures of many bark beetles have been described [13,15,16,17,18,19] and the ecological roles of stridulation in some bark beetle species are well studied [18,20,21,22,23,24,25,26,27]. Interestingly, bark beetle stridulation sounds influence pheromone production and composition of bark beetle partners within the tree. For example, *Dendroctonus* bark beetles that stridulate near the entrance hole of a potential partner stimulates the beetle within the tunnel to alter the chemical composition or even stop production of their aggregation pheromone [28,29].

Pheromone communication has been a primary focus of bark beetle management [30,31,32] due to its many successes [33,34,35,36,37]. The pheromone composition of most tree-killing bark beetles is known [38,39,40,41,42] and synthetic pheromone products for these species are available for monitoring, tree protection, and disruption [43]. Management methods such as tree protection, monitoring, and disruption targeting acoustic or vibratory communication in bark beetles have been proposed [32,44,45] but not yet utilized in the field. Laboratory studies using vibroacoustic playback treatments that include recordings of bark beetles, wood borers, or artificial sounds have shown promise in reducing beetle entry, tunneling behavior, and egg laying of two tree-killing *Dendroctonus* species in logs [44,45,46] but appears to be less effective against secondary bark beetles, such as *Ips pini* (Say) [46]. However, stridulation by *Ips* bark beetles is important in their ecology as female beetles stridulate when entering another beetle’s gallery. Male *Ips* beetles will not allow a female to enter his gallery unless she stridulates [11,13,47]. Female *Ips* also stridulate when stressed or attacked (e.g., held in the mandibles of a predatory insect) [47,48,49], during gallery construction [11,50], interactions with other beetles within the gallery [51], and prior to mating to call the resident male beetle [47,48]. Sivalinghem [17] found that the characteristics of the stridulation sounds differed depending on the context and situation. As male *Ips* do not stridulate, male–male confrontations are not associated with stridulation, as seen in interactions among male *Dendroctonus* beetles [18,45].

Why vibroacoustic treatments are not effective against *Ips* bark beetles is unclear, given the importance of stridulation in many aspects of their ecology [11,52]. Aflitto & Hofstetter [46] provide two suggestions for the lack of response by *Ips*: (1) differences in life history traits compared to *Dendroctonus* beetles, such as the sex that initiates colonization, which is the female for *Dendroctonus* and the male for *Ips*, or (2) differences in their ability to perceive vibrations or airborne sounds. For instance, the frequencies or amplitudes administered by the tactile transducer in their study might not be perceived by *Ips* beetles. To further test the second assumption, we propose additional vibroacoustic treatments that expose *Ips* beetles to a larger array of frequencies and patterns than that tested by Aflitto & Hofstetter [46]. We use the pinyon engraver *Ips confusus* LeConte that colonizes living pinyon trees as our model system.

Acoustic communication is present in at least half of the subtribes of the Scolytinae, and stridulatory structures of bark beetles have evolved multiple times [52]. Three primary stridulatory mechanisms within bark beetles (Scolytinae) are known: elytro-tergal, vertex-pronotal, and gula-prosternal stridulatory organs [11,12,45]. Stridulatory structures of *Ips confusus* have been described by Barr [11] but the stridulation sounds produced by female *I. confusus* have not been analyzed or illustrated [12]. *Ips confusus’* stridulatory structure is categorized as vertex-pronotal type [11]. To allow for vertex-pronotal stridulatory organs, the morphological structures of connective tissues between the head and pronotum of *Ips* beetles are different from other bark beetle genera that do not stridulate this way (described by Barr [11]).

How bark beetles perceive vibrations and airborne sounds is unknown, and an ‘ear’ has yet to be found in bark beetles [45]. Sounds and vibrations propagate through wood via mechanical waves, and the type and location of the stridulatory organs likely play a role in how signals are used and transmitted [53,54]. For instance, bark beetle stridulatory signals could provide both airborne and substrate-borne information that is perceived by neighboring beetles within the tree. Bark beetle stridulatory signals are broadband, pulsatile, with most energy focused at intermediate frequencies (from 3–12 kHz) which are promising signals for bimodal (vibrations and airborne) acoustic communication as low frequency components may travel far within the tree while airborne signals may remain limited to tunnels within the tree.

The objectives of this paper were to (1) describe the stridulation apparatus and acoustic stress chirps of *Ips confusus*, and (2) test whether vibroacoustic treatments within tree tissues affect entry, mating, and fecundity of *I. confusus*. Using a tactile transducer device, which allows for efficient input of vibroacoustic energy into wood, we tested whether vibroacoustic signals, both natural and unnatural, discourage bark beetle entry, disrupt male–female interactions, or progeny production within galleries. The ultimate goal of the second objective was to test the efficacy of off-the-counter acoustic devices for bark beetle control and advance management options that could expand the arsenal of tools that land managers use to control *Ips* bark beetles.

## 2. Materials and Methods

### 2.1. Collection of Insects and Tree Materials

Live *Ips confusus* adults were collected from May to October in 2020 from naturally colonized single-leaf pinyon pine (*Pinus monophylla*) located northeast of Flagstaff, Arizona, USA (35°25′ N, 111°33′ W; 1987 m asl) in pinyon–juniper woodland habitat. Beetle-infested tree materials were placed into emergence cans in the laboratory (20–25 °C, 50–60% humidity) and adult beetles were collected upon emergence. Species and sexual identification of *I. confusus* were conducted according to Wood [55] with additional confirmation of females by the presence of vertex stridulatory structure on the head. Voucher specimens are stored at the Forest Entomology laboratory of R. Hofstetter in the School of Forestry, Northern Arizona University. Five healthy, uninfested *P. monophylla* at a location 25 miles south of Flagstaff, AZ (35°04′ N, 111°24′ W; 2119 m asl) were cut into 60 cm logs for behavioral response trials (see Section 2.4) on May 2020.

### 2.2. Stridulation Apparatus Anatomy

**Figure 1:**
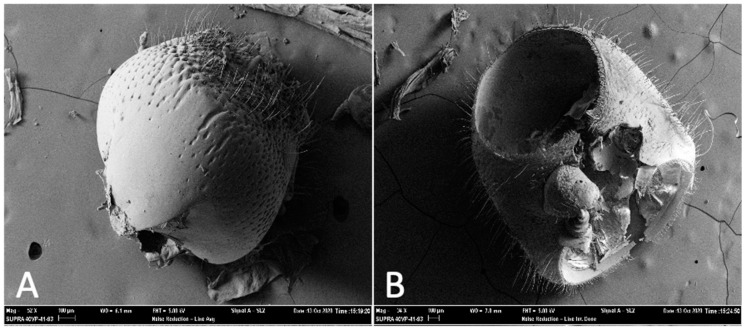
Scanning electron images of head and thorax of *Ips confusus* adult male. (**A**) Posterior dorsal view of head (52× magnification) and (**B**) pronotum (34× magnification). No stridulatory structures present. See Figure 2 for comparison of stridulatory structures on female head and thorax.

**Figure 2:**
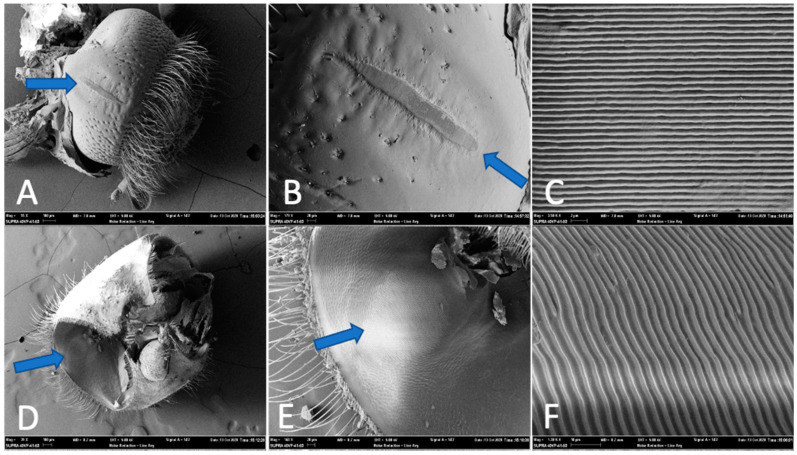
Scanning electron images of stridulatory structures of *Ips confusus* adult female. (**A**) Posterior dorsal view of head with plectrum viewed at center of image (55× magnification), (**B**) close up of entire plectrum on head (179× magnification), (**C**) ridges of plectrum on head (3500× magnification), (**D**) view of file at the anterior section of pronotum; circular and similar to a fingerprint (39× magnification), (**E**) close up of file, underside of pronotum (160× magnification), and (**F**) ridges of file on pronotum (1300× magnification).

To examine the stridulation apparatus of *Ips confusus*, the head and thorax of three male and three female adults were prepared, cleaned, and sputter coated with gold for 60 s using a DESK II Dentron Vacuum unit. Specimens were examined using a Zeiss Supra 40VP scanning electron microscope (SEM) at Northern Arizona University. Stridulatory structures from SEM images were measured digitally using Zeiss SMART SEM software. Male *I. confusus* exhibited no vertex-pronotal stridulatory structures and thus no measurements were recorded for male specimens (but an image is provided, Figure 1). All stridulatory measurements refer to structures found on adult female beetles. The *pars stridens* (structure on head) width and length, the total number of ridges, and the average distance between a subset of ridges were measured from three females (Figure 2A–C). The *plectrum* (structure inside pronotum) width and length, the total number of ridges, and the average distance between a subset of ridges were measured for the same three females (Figure 2D–F).

### 2.3. Airborne Acoustic Signals

Distressed sounds produced by *Ips confusus* adults were recorded and elicited by gently holding the beetle by the abdomen with soft forceps (featherweight forceps #4748, BioQuip) to allow for full range of movement of the vertex-pronotal structures. Of the bark beetles that produce sounds, most stridulate in response to disturbance such as predation or being held. In this paper, we call these sounds ‘stress chirps’ [18,23,56,57]. Out of 29 recorded females, 17 females produced sounds that were of sufficient quality for acoustic analyses. All recordings were performed holding the beetle 20 mm from an ultrasonic electronic insertion microphone (developed by Dunn [58] as reported in Yturralde & Hofstetter [19]). The microphone was constructed using a Knowles Acoustics FG-3329 electret condenser microphone (Knowles Electronics, Itasca, IL, USA), which is sensitive to frequencies between 10 Hz and 10 kHz at −53 dBV/0.1 Pa and linearly declines above 10 kHz such that at 48 kHz sensitivity is approximately −70 dBV/0.1 Pa (Knowles Electronics 2005, 2013). However, this microphone is reported to detect frequencies in excess of 100 kHz [58,59]. Airborne acoustic signals were recorded using an HD-P2 TASCAM digital audio recorder at 96 kHz and 24 bit sampling rate.

**Figure 3:**
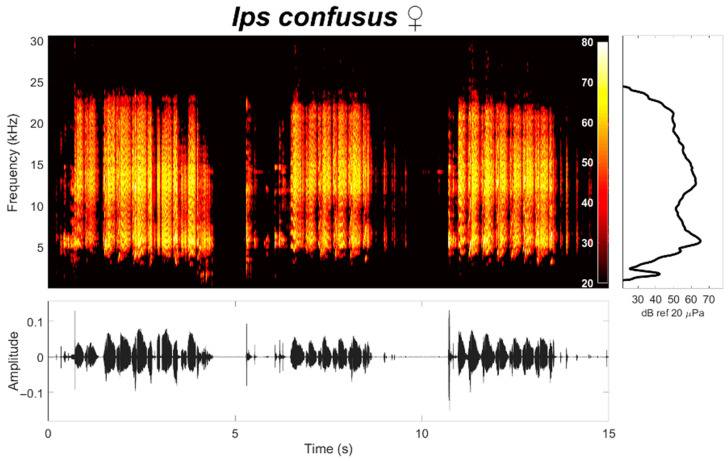
Spectrogram (top), waveform (bottom), and mean spectrum (right) of stridulations produced by female *Ips confusus* when held with forceps. Male *I. confusus* do not have stridulatory organs and did not stridulate.

Recordings of 17 female beetles were automatically analyzed using feature extraction methods developed in MatLab R2018b [12]. Spectrograms were produced using the following parameters: Hamming window with a 1024 sample size, 1024 frequency bins, and 75 % overlap (768 samples) (Figure 3). Dominant frequency, centroid frequency, minimum frequency, maximum frequency, chirp duration, number of syllables within a chirp, note duration, and strikers per syllable were automatically estimated. Note refers to an individual stridulation sound, while a train of syllables is referred to as a chirp. Reported spectral characteristics were estimated following the procedures of Bedoya et al. [12].

### 2.4. Vibroacoustic Treatments in Logs

#### 2.4.1. Assay 1 Effects of Vibroacoustic Treatments on Male *I. confusus* Entry into Logs

Freshly cut, un-infested single-leaf pinyon pine (*Pinus monophylla*) logs, averaging 60 cm (±1 cm) long and 15 cm (±3 cm) diameter rested on foam (4 cm wide polystyrene foam board, Home Depot) placed on a table in the lab (~25 °C, ~50% humidity) with natural light coming from windows. An exciter (Dayton Audio DAEX25FHE-4 Framed High-Efficiency 25 mm Exciter 24 W 4 Ohm) was attached to the xylem at the bottom cut (i.e., lower in the tree) of each log, approximately halfway between the center point and the edge (where the bark attaches). The exciter conducts vibrating energy into the wood (i.e., xylem) surface, allowing the log to radiate vibroacoustic energy as though it was a speaker. The exciter uses the inertia of its own physical mass to apply force from the voice coil to the xylem surface sending vibrations through the log.

Vibroacoustic treatments were initiated using a digital player amplifier (Lepai LP-269 FS, 45 W × 4 R.M.S.) and mp3 files (MPEG-2 Audio encoded data at 32-bit, 48 kHz, 224 kbps CBR; Adobe Audition Pro) stored on a memory card. We recognize the conversion to mp3 files results in the loss of some acoustic features due to compression and encoding algorithms and thus used a high bit rate of 224 kbps [60,61]. These file formats were specifically chosen to represent typical audio outputs of commercial products available to land managers and owners.

**Figure 4:**
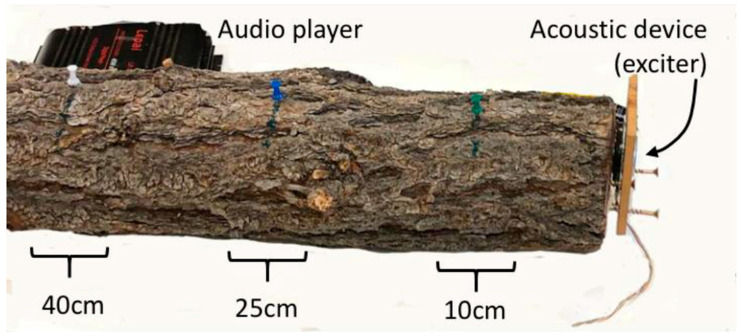
Acoustic treatment assay setup. Pinyon log with the labeled distances: 10 cm (green pin), 25 cm (blue pin), 40 cm (white pin) where beetles were introduced. Placement of acoustic device at end of cut log. Audio player (dark object) also in image.

Newly emerged male *I. confusus* were each placed into drilled holes (5 mm dia., phloem depth) at three distances (10 cm, 25 cm, and 40 cm) from the bottom cut of the log where the exciter was placed (Figure 4). At each of these distances, five males were introduced, approximately 5 cm spacing around the log, into the phloem and sealed using half of a gel capsule (size 0, clear vegetarian capsule; Capsuline^®^, Dania Beach, FL, USA).

**Figure 5:**
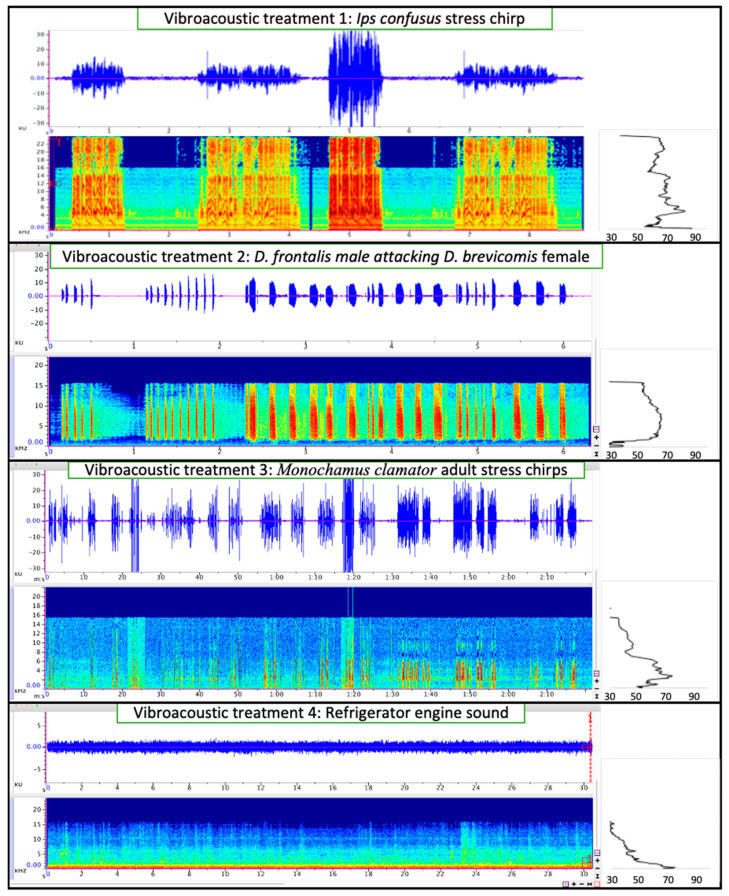
Waveform (top panel, amplitude over time in seconds, spectrograph (bottom panel, kHz over time in seconds) and mean spectrum (right panel, kHz over dB re 20 μPa) of vibroacoustic treatments (1 through 4) played into logs to test *Ips confusus* behavior. The *x*-axis is in seconds.

**Figure 6:**
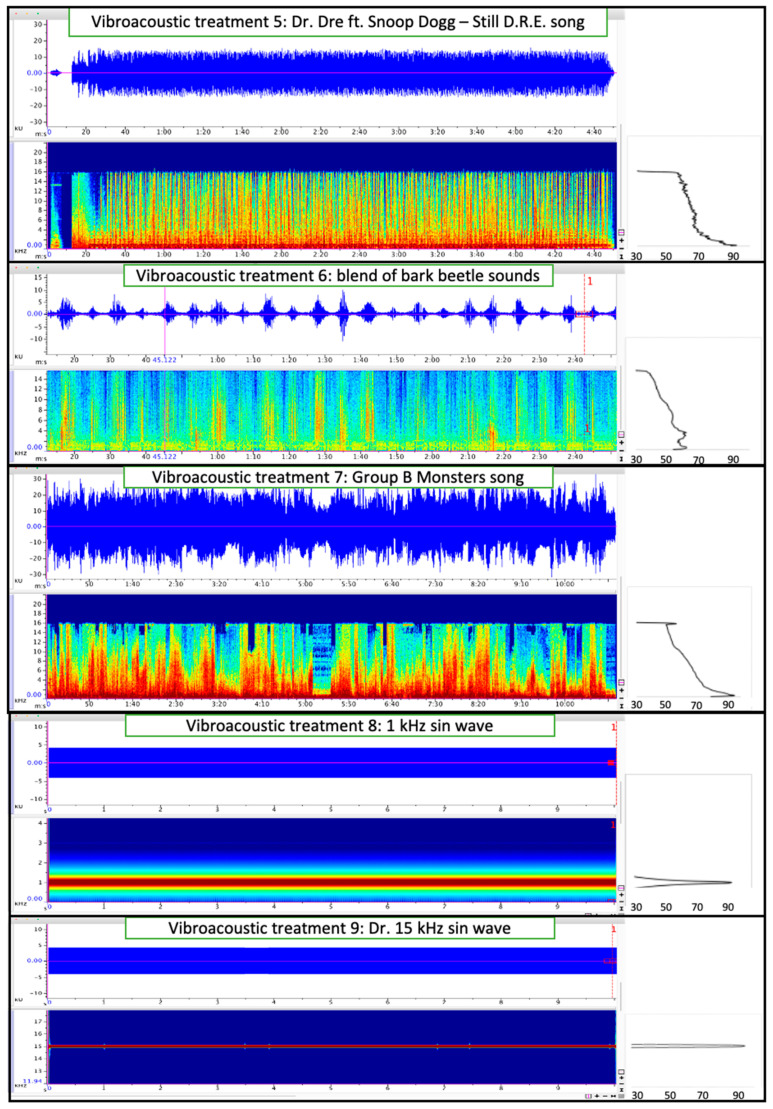
Waveform (top panel, amplitude over time in seconds), spectrograph (bottom panel, kHz over time in seconds), and mean spectrum (right panel, kHz over dB re 20 μPa) of vibroacoustic treatments (5 through 9) played into logs to test *Ips confusus* behavior. The *x*-axis is in seconds.

Once placed in the log, male beetles were monitored to determine entry into the phloem—as evidenced by frass in the gel capsule. Nine vibroacoustic treatments (one vibroacoustic treatment per log) were tested (Figure 5 and Figure 6) over eight days. A no-vibroacoustic control log with the same number of male beetles was performed each time a set of vibroacoustic treatments were initiated. Vibroacoustic treatments included: (1) *Ips confusus* stress chirps produced by female beetles, (2) *Dendroctonus frontalis* Hopkins (southern pine beetle) aggression call produced when confronting female *D. brevicomis* in gallery [44], (3) *Monochamus clamator* LeConte 1852 (spotted pine sawyer woodborer) adult distress call [46], (4) audio recording of refrigerator engine [62] (5) musical song ‘Dr. Dre ft. Snoop Dogg—Still D.R.E.’, (6) blend of bark beetle stridulation sounds that include attraction, distress and aggression chirps from three *Dendroctonus* species [44], (7) musical song ‘Group B monsters—with pure engine sounds’, (8) 1 kHz sin wave (created by Adobe Audition 2020), and (9) 15 kHz sin wave (Adobe Audition 2020). In total, 180 males were placed into logs: 15 beetles for each of the nine vibroacoustic treatments and 15 beetles (×3) for control logs. Vibroacoustic treatments, such as treatments 1, 2, 3, and 6, were selected based on effects observed with other beetle species [44,46] while additional treatments such as music and sin waves were selected as additional “negative” controls to show that most sounds are not likely to affect beetle behavior [45]. Beetles were introduced at one of three distances on the log from the vibroacoustic device: 10 cm, 25 cm, and 40 cm. After 48 h, males that tunneled into the phloem and produced abundant frass were recorded as ‘entry’, and males that remained in the gel capsule and produced little or no frass were recorded as ‘no entry’. Beetles will typically tunnel immediately into the tree if it is suitable [5]. We selected a 48 h time limit so that beetles were not forced to chew into the wood to either escape or find substance to survive. This design, with all beetles within a treatment in the same log, allowed us to remove host tree effects which can be significant and limit the number of vibroacoustic players and log samples needed. We acknowledge the limits of statistical inference and potential nesting effects using this design.

**Figure 7:**
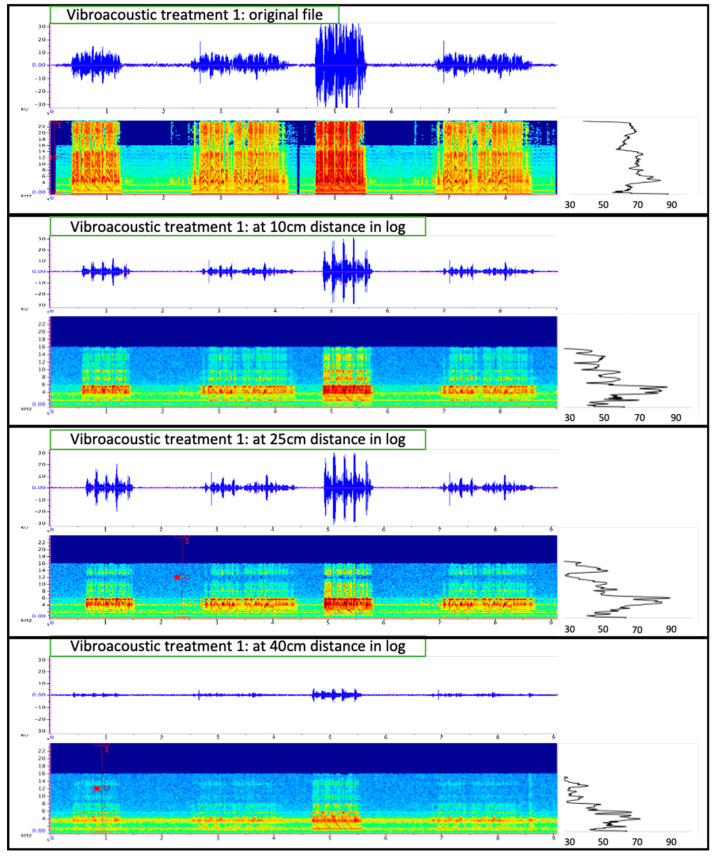
Waveform (top panel: amplitude over time in seconds), spectrograph (bottom panel: kHz over time in seconds) and mean spectrum (right panel, kHz over dB re 20 μPa) of vibroacoustic Treatment 1 (*Ips confusus* stress call) of original vibroacoustic file (top image set) and recordings of vibroacoustic input in the log at 10 cm, 25, and 40 cm from the speaker.

Treatment within logs were re-recorded at each of the three distances (10 cm, 25 cm, and 40 cm) to observe what beetles experience within the log at that distance from the exciter (i.e., speaker) (Figure 7). Vibroacoustic outputs were re-recorded using two methods: (1) needle-piezo element receiver and (2) laser vibrometer. For method one, we used a HD-P2 TASCAM digital audio recorder at 96 kHz and 24 bit sampling rate with a microphone consisting of a piezo element (35 mm ceramic, part # MCBT-457-RC, 2.8 kHz resonant frequency, 600 Ω resonant impedance max.) glued to a 2 cm diameter maple wood dowel with a steel phonograph needle (0.90 mm diameter, 15.63 mm long). The needle was placed into the phloem reaching into the xylem at a depth of 5 mm. The needle-piezo element effectively recorded vibroacoustic energy within the phloem and xylem tissues at the appropriate location where beetles tunneled. Our second method used a Polytec OFV-500 Vibrometer with an OFV-534 sensor head using a Keysign MSOX41044 mixed-signal oscilloscope to measure playback outputs at the three distances in logs. The vibrometer sensor focused on a point on reflective tape placed on xylem surfaces within each entry hole but because the reflective tape did not connect well to the rough xylem surface where beetles were introduced, the laser vibrometer data were not reliable or accurate and thus not presented.

#### 2.4.2. Assay 2 Effects of Vibroacoustic Treatments on *I. confusus* Mating, Tunneling, and Fecundity

To test the effects of vibroacoustic treatments on disruption of mating and egg laying, a male *I. confusus* was introduced into the log (with a gel capsule) and a female beetle was introduced 48 h later (in the gel capsule). Once female beetles were introduced, the same vibroacoustic treatments as described and tested in Assay 1 were initiated. A total of 15 pairs (male and female beetles) were monitored per log to determine if the vibroacoustic treatment affected female entry, gallery length, egg laying, and adult survival. One female was added to each male via the gel capsule. Each pair was monitored by observing evidence of additional frass and presence of live or dead beetles in the gel capsule for 12 days. Vibroacoustic treatments were played continuously throughout the experiment once females were added to the log. After 12 days, the bark was carefully stripped from the logs and the following data were collected for each beetle pair: male condition (alive, dead or absent), female condition (alive, dead, or absent), female oviposition tunnel length, and characteristics (straight, curved, irregular, or absent), and number of progeny (eggs and larvae).

### 2.5. Statistical Analysis

For statistical analysis, all data were checked for normality (Shapiro–Wilk test) and homogeneity of variance (Levene’s test). In a case when both previously mentioned assumptions were satisfied, a one-way ANOVA was used, with a Tukey HSD test as post hoc. In a case when homogeneity of variance was violated, Welch’s ANOVA test was used, with a Tukey HSD test post hoc. When normality and homogeneity of variance was violated, nonparametric Kruskal–Wallis ANOVA test was conducted including a correction for multiple tests, together with the post hoc multiple comparisons of mean ranks test. A generalized linear mixed model with a logistic link function was also used to test distance from the speaker on beetle entry rate (R ver. 3.6.2, Vienna, Austria). A negative binomial generalized linear model (GLM) was used to assess differences in tunnel length and progeny per beetle pair across distances and vibroacoustic treatments. All test significance levels were set at *p* < 0.05.

## 3. Results

### 3.1. Stridulation Apparatus Anatomy

Female *Ips confusus* possessed vertex-pronotal structures (Figure 2) that are consistent with other *Ips* species [11,13,63]. Stridulatory organs consisted of a *plectrum* on the underside of the anterior dorsal part of the pronotum (Figure 2D–F) and a *pars stridens* located on the posterior dorsal part of the head (Figure 2A–C). The *plectrum* was 237.6 ± 10.2 um wide and 297.1 ± 22.8 um long and consists of a circular set of ridges (like a fingerprint), with approximately 92 ± 9 ridges at 842 ± 14 ridges/mm. Average distance between ridges of the *plectrum* was 3.378 ± 0.118 um. Some ridges were bifurcated. Prior to gold coating for the SEM, the entire *plectrum* appeared to be flexible, with little sclerotization (i.e., the structure is semitransparent). The *pars stridens* (file on the head) is long (380.2 ± 15.0 um) and narrow (45.7 ± 3.0 um at its widest) and contained 475 ± 23 ridges (1567 ± 29 ridges/mm), with an average of 801.5 ± 7.5 nm distance between each ridge top to ridge top. Male *I. confusus* adults did not possess vertex-pronotal structures (Figure 1).

### 3.2. Acoustic Characteristics of Female Ips confusus

Female *I. confusus* produced sounds similar to those described by other *Ips* species when in distress such as when held by a predator or grabbed by forceps. Stridulations of *I. confusus* consisted of a series of broadband sounds emitted in groups (chirps) separated by a period of silence (inter-chirp interval) (Figure 3). Duration of each chirp in response to disturbance ranged from 0.45 s to 4.32 s with a mean of 2.00 ± 1.02 s (SD) and an inter-chirp interval (i.e., no sound) of 3.69 ± 1.78 s (mean ± SD). Each chirp consisted of a series of 9.84 (±5.22 SD) syllables (with 100–200 strikes per syllable) with the following parameters (mean ± SD, N = 17, *n* = 532): dominant frequency (12.47 ± 5.87 kHz), centroid frequency (13.45 ± 4.96 kHz), minimum frequency (5.99 ± 2.43 kHz), max frequency (22.19± 6.68 kHz), note duration (0.21 ± 0.37 s), inter-note interval (0.08 ± 0.06 s), note rate (5.23 ± 1.95 syllables per second) and strike rate (1702 ± 919 strikes per second; range of 769 to 4000 strikes per second). *Ips confusus* males did not produce sounds when held, other than those indirectly associated with moving legs, wings, or mandibles.

### 3.3. Behavioral Response of Vibroacoustic Treatments in Logs

#### 3.3.1. Assay 1: Effects of Vibroacoustic Treatments on Male *I. confusus* Entry into Logs

Vibration/sound amplitude decreased with distance and some of the upper frequencies or low amplitude vibroacoustic energy were lost with distance from the exciter (e.g., Figure 7). Additionally, vibroacoustic treatment output was limited to 16 kHz due to limits of the audio equipment used in the study.

Male *I. confusus* adult entry rates into logs were not statistically different than the control logs (no vibroacoustic treatment) (Kruskal–Wallis test: H > 14.75, d.f. = 11, *p* > 0.19) and thus entry was not affected by vibroacoustic treatments. Ninety-eight percent of male beetles entered into the control logs and 99% of beetles entering into vibroacoustic treatment logs. Distance of the entry hole (set at 10 cm, 25 cm, and 40 cm) from log edge, where exciter was placed, had no effect on male entry levels, regardless of vibroacoustic treatment (all *p* > 0.10).

#### 3.3.2. Assay 2: Effects of Vibroacoustic Treatments on *I. confusus* Mating, Tunneling, and Fecundity

**Figure 8:**
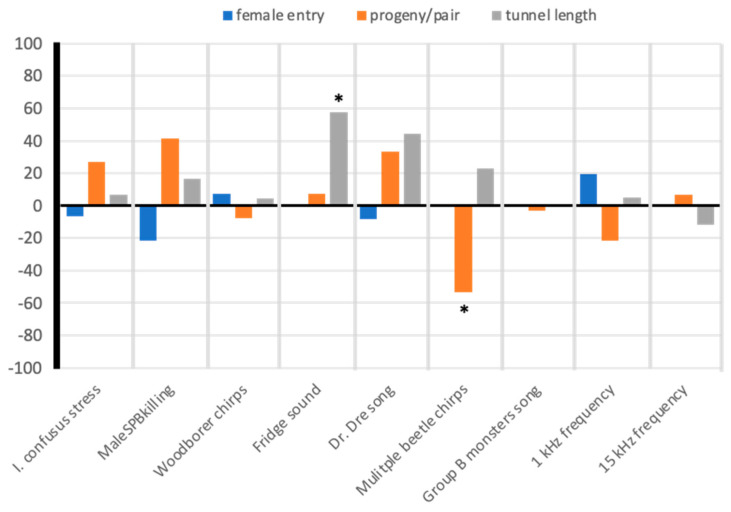
Relative effect (%) of acoustic treatments compared to controls (no vibroacoustic input) on female *Ips confusus* entry into male gallery hole (blue bars), progeny per beetle pair (orange bars) and tunnel length (created by female beetle; grey bars). A positive value means that beetles performed better in the acoustic treatment log than the control log, and a negative value mean that beetles performed poorer in the acoustic treatment log than in the controls log. * indicates a slight significant (*p* < 0.10) difference from the control.

Female *I. confusus* adult entry rates into galleries with a male were not statistically different than the control treatment and thus female entry was not affected by vibroacoustic treatments (Table A1, Table A2 and Table A3 in Appendix A; Figure 8, blue bars). Female entry ranged from 67% to 100% in logs but did not statistically differ across vibroacoustic treatments. Female tunnel lengths at 12 days differed slightly between one of the vibroacoustic treatments compared to the controls (Figure 8 grey bars; Table A2); beetle pairs had longer tunnels in the audio recording of refrigerator engine vibroacoustic treatment than the control. Progeny per beetle pair differed slightly between one of the vibroacoustic treatments and the control treatment (Figure 8 orange bars; Table A2); progeny per beetle was lower in the ‘multiple beetle chirps’ vibroacoustic treatment compared to the control. Distance of entry point from vibroacoustic device did not affect female entry, tunnel length, or progeny per pair (*p* > 0.10 for all distances and dependent variables).

## 4. Discussion

### 4.1. Stridulatory Structures

Stridulatory structures of female *Ips confusus* are a similar shape to other *Ips* species that have a vertex-pronotal stridulatory apparatus [15,17,47,64]. The number of ridges and the length and width of the *plectrum* found on *I. confusus’* pronotum are within the same range of *I. pini* and *I. plastographus*. The *pars stidens* is also similar in shape to other *Ips* species, except that the length of the *pars stridens* on *I. confusus* is longer by more than 100 μm (~30% longer) than *I. pini* and *I. plastographus*. Number of ridges in the *pars stridens* also differs from published literature in that we found approximately twice as many ridges on *I. confusus* compared to *I. pini* measured by Sivalinghem [17] and *I. plastographus* measured by Oester [64] (but half the number of ridges on *I. confusus* compared to *I. pini* measured by Swaby and Rudinsky [15]).

### 4.2. Stridulation in Response to Disturbance

Most females stridulated immediately upon being grabbed by forceps. Females often stridulated for 1 to 4 s and then were quiet until grabbed again. Females were also observed under the microscope to stridulate when placed on their back. Thus, the movement associated with stridulation can be used to distinguish between live male from female beetles, as males did not exhibit these movements. Most bark beetle species, including *I. confusus*, are reported to generate ‘distress’, ‘stress’, or ‘disturbance’ chirps [11,14,18,57]. Features of stress chirps varied significantly within and between females, as seen with *I. pini* females [17,47,65] and *I. plastographus* females [64]. Significant differences between the temporal and amplitude characteristics of *Ips* chirps have been observed in different contexts [47,51], and it is also likely that characteristics of *I. confusus* chirps are context-dependent. Female size likely influences signal characteristics [17,19] in addition to female condition. Temporal patterns (length of chirp, number of strikers per chirp) of stress chirps produced by *I. confusus* and other *Ips* species appear to be more variable than stress chirps produced by *Dendroctonus* bark beetles [18,19].

Duration of stress chirps of *I. confusus* (e.g., 2 s on average) are longer than that of female *I. pini* (e.g., 0.81 s, Swaby and Rudinsky [15]; 0.16 s, Sivalinghem [17]) but similar to *I. plastographus* [15]). Longer chirp lengths by *I. confusus* compared to *I. pini* may result from their longer pars stridens rather than strike rate which was similar to the stress chirp reported for *Ips pini* [15]. The dominant frequency of stress chirps by *I. confusus* varied significantly across chirps and among beetles and is lower in frequency than the distress chirps of *I. pini* [17] but similar to *I. pini* chirps in other contexts such as male–female interactions [17]. Lower frequency of *I. confusus* is likely a result of their slightly larger body size compared to *I. pini* (3.3–4.3 mm length of *I. pini* [55] vs. 4.05–4.57 mm length of *I. confusus*; beetles measured in our study) or strike intensity. Larger bark beetles typically have chirps with lower dominant frequencies [19].

### 4.3. Effects of Vibroacoustic Treatments on Beetle Entry, Gallery Length, and Progeny Production

Eriksson et al. [66] used vibrational signals to disrupt mating in leafhoppers, illustrating the potential for acoustic management of insect pests on plants. By transmitting vibrations through grapevine plants, they were able to reduce the searching success of male leafhoppers and complete cessation of communication with females [67]. Early attempts to use acoustics for manipulating bark beetle behavior have been mixed, depending on the bark beetle genus. Hofstetter et al. [44] were able to alter tunneling behavior and reduce egg laying in logs by *Dendroctonus* species with vibroacoustic treatments introduced into the logs using exciters (as performed in this study). However, Aflitto and Hofstetter [46] were unable to significantly influence entry or tunneling of *Ips pini* within logs but did reduce the entry of *Dendroctonus* beetles into logs using their stress chirps. Our study found that vibroacoustic treatments generally had no effect on *I. confusus* male entry, but had variable effects on male–female acceptance, female tunneling, or progeny per pair. Only vibroacoustic treatment with multiple bark beetle chirps had a significant negative effect on female beetles (i.e., reduced progeny). Some vibroacoustic treatments resulted in a slight increase in tunneling length but these treatments did not affect progeny production or entry behavior. Given that *I. confusus* produces sounds, it is likely that they hear and respond to particular sounds and vibrations in their environment whether from their partner, potential rivals, or predators [17,68]. The conversion of recordings to mp3 for playbacks could have affected vibroacoustic components [59,61] that could have influenced beetle responses to particular treatments. Also, based on re-recordings of the vibroacoustic treatments within the log, the amplitude decreased with distance, particularly between 25 and 40 cm from the exciter (see Figure 7) and only vibroacoustic waves below 16 kHz were present in the log.

Bark beetle communication is multimodal [5,45], involving long and short-range pheromones, short-range acoustic signals (as discussed in this paper), and contact chemical signals [69] that are important when in the tree [5]. *Ips confusus* in our study may be able to overcome the disruption caused by the vibroacoustic input by focusing on other modes of communication, particularly chemical pheromones or contact chemicals. Suggestions for causes for the lack of significant effects of vibroacoustic on *I. confusus* are that (1) the vibroacoustic input through the wood is an amplitude, duration, or frequency that would not affect or disrupt behavior, (2) the vibroacoustic treatments work initially but are then ignored as the insect habituates to the vibroacoustic inputs, or (3) the selected treatments do not affect insects in a manner that disrupts the particular behaviors we measured (entry, reproduction, tunneling, or egg laying).

## 5. Conclusions

We described the stridulatory organs and ‘stress’ chirp of the pinyon engraver, *Ips confusus*. Only female *I. confusus* stridulate using a vertex-pronotal structure. No stridulatory organ occurs on adult male *I. confusus*. Females produced stress chirps but chirp duration, pulses per chirp, and dominant frequency varied significantly within and across beetles. We tested a vast array of vibroacoustic treatments into logs to try to disrupt male entry into logs, as well as female–male interactions, female tunneling, and female oviposition. We found that none of these vibroacoustic treatments affected male *I. confusus* entry into logs and vibroacoustic treatments had varied effects on female behavior. We suggest further studies if vibroacoustic methods are to be used for *Ips confusus* control.

## Data Availability

The data presented in this study are available upon request from the corresponding author. The data are not publicly available because of a potential patent.

## Appendix A

**Table A1:** Post hoc statistical analysis (Tukey HSD test) of measured variables (Mean ± SD) between acoustic treatments. Letter “a” represents statistically not significant difference between acoustic treatment groups or lengths on the log (female tunnel lengths: Welch’s ANOVA–F_11, 15.18_ = 2.789, *p* = 0.033 *; number of progeny per beetle pair: one-way ANOVA: F_1,11_ = 1.52, *p* = 0.162).

Acoustic Treatment	Measure Type	Mean ± SD
*Ips confusus* stress chirps produced by female beetles ~10 cm	Female tunnel lengths	25.35 ± 12.86 a
	Number of progeny per beetle pair	9.50 ± 0.75 a
*Ips confusus* stress chirps produced by female beetles ~25 cm	Female tunnel lengths	36.75 ± 7.80 a
	Number of progeny per beetle pair	9.90 ± 1.46 a
*Ips confusus* stress chirps produced by female beetles ~40 cm	Female tunnel lengths	20.60 ± 19.03 a
	Number of progeny per beetle pair	9.12 ± 3.11 a
*Dendroctonus frontalis* Hopkins (southern pine beetle) aggression call produced when confronting female *D. brevicomis* in gallery ~10 cm	Female tunnel lengths	30.60 ± 9.07 a
	Number of progeny per beetle pair	10.04 ± 1.88 a
*Dendroctonus frontalis* Hopkins (southern pine beetle) aggression call produced when confronting female *D. brevicomis* in gallery ~25 cm	Female tunnel lengths	30.33 ± 7.23 a
	Number of progeny per beetle pair	9.56 ± 1.33 a
*Dendroctonus frontalis* Hopkins (southern pine beetle) aggression call produced when confronting female *D. brevicomis* in gallery ~40 cm	Female tunnel lengths	35.00 ± 10.39 a
	Number of progeny per beetle pair	11.76 ± 1.20 a
*Monochamus clamator* LeConte 1852 (spotted pine sawyer woodborer) adult distress call ~10 cm	Female tunnel lengths	18.80 ± 12.65 a
	Number of progeny per beetle pair	8.94 ± 3.74 a
*Monochamus clamator* LeConte 1852 (spotted pine sawyer woodborer) adult distress call ~25 cm	Female tunnel lengths	15.00 ± 6.00 a
	Number of progeny per beetle pair	7.42 ± 1.26 a
*Monochamus clamator* LeConte 1852 (spotted pine sawyer woodborer) adult distress call ~40 cm	Female tunnel lengths	28.20 ± 14.35 a
	Number of progeny per beetle pair	11.52 ± 4.13 a
Control ~10 cm (no vibroacoustic treatment)	Female tunnel lengths	21.40 ± 11.10 a
	Number of progeny per beetle pair	8.64 ± 3.77 a
Control ~25 cm (no vibroacoustic treatment)	Female tunnel lengths	17.40 ± 12.28 a
	Number of progeny per beetle pair	7.50 ± 1.97 a
Control ~40 cm (no vibroacoustic treatment)	Female tunnel lengths	30.00 ± 14.49 a
	Number of progeny per beetle pair	10.90 ± 0.72 a

**Table A2:** Post hoc statistical analysis (Tukey HSD test) of measured variables (Mean ± SD) between acoustic treatments. Letter “a” represents a statistically not significant difference between acoustic treatment groups or lengths on the log (female tunnel lengths: Welch’s ANOVA–F_11, 15.18_ = 1.42, *p* = 0.208; number of progeny per beetle pair: Kruskal–Wallis ANOVA: H = 23.80, d.f. = 11, *p* = 0.016 *).

Acoustic Treatment	Measure Type	Mean ± SD
Audio recording of refrigerator engine ~10 cm	Female tunnel lengths	22.75 ± 9.21 a
	Number of progeny per beetle pair	10.05 ± 2.34 a
Audio recording of refrigerator engine ~25 cm	Female tunnel lengths	49.75 ± 18.80 a
	Number of progeny per beetle pair	15.60 ± 4.14 a
Audio recording of refrigerator engine ~40 cm	Female tunnel lengths	40.25 ± 29.60 a
	Number of progeny per beetle pair	11.80 ± 4.61 a
Musical song ‘Dr. Dre ft. Snoop Dogg-Still D.R.E.’ ~10 cm	Female tunnel lengths	50.00 ± 9.82 a
	Number of progeny per beetle pair	12.86 ± 2.83 a
Musical song ‘Dr. Dre ft. Snoop Dogg-Still D.R.E.’ ~25 cm	Female tunnel lengths	48.00 ± 5.29 a
	Number of progeny per beetle pair	11.06 ± 0.80 a
Musical song ‘Dr. Dre ft. Snoop Dogg-Still D.R.E.’ ~40 cm	Female tunnel lengths	42.00 ± 33.53 a
	Number of progeny per beetle pair	10.07 ± 3.87 a
Blend of bark beetle sounds that include attraction, distress and aggression chirps from three *Dendroctonus* species ~10 cm	Female tunnel lengths	22.50 ± 8.81 a
	Number of progeny per beetle pair	10.82 ± 1.06 a
Blend of bark beetle sounds that include attraction, distress and aggression chirps from three *Dendroctonus* species ~25 cm	Female tunnel lengths	18.50 ± 7.18 a
	Number of progeny per beetle pair	10.50 ± 3.60 a
Blend of bark beetle sounds that include attraction, distress and aggression chirps from three *Dendroctonus* species ~40 cm	Female tunnel lengths	11.00 ± 10.14 a
	Number of progeny per beetle pair	8.96 ± 3.07
Control ~10 cm (no vibroacoustic treatment)	Female tunnel lengths	43.50 ± 3.53 a
	Number of progeny per beetle pair	14.00 ± 1.13
Control ~25 cm (no vibroacoustic treatment)	Female tunnel lengths	29.75 ± 24.68 a
	Number of progeny per beetle pair	9.90 ± 3.36 a
Control ~40 cm (no vibroacoustic treatment)	Female tunnel lengths	4.80 ± 10.73 a
	Number of progeny per beetle pair	8.50 ± 4.53 a

**Table A3:** Post hoc statistical analysis (multiple comparisons of mean rank tests) of measured variables (means ± SD) between acoustic treatments. Letter “a” represents a statistically not significant difference between acoustic treatment groups or lengths on the log (female tunnel lengths: Kruskal–Wallis ANOVA–H (11) = 8.89, *p* = 0.631; number of progeny per beetle pair: Kruskal–Wallis ANOVA–H (11) = 10.84, *p* = 0.456).

Acoustic Treatment	Measure Type	Mean ± SD
Musical song ‘Group B monsters—with pure engine sounds’ ~10 cm	Female tunnel lengths	26.00 ± 16.91 a
	Number of progeny per beetle pair	10.82 ± 4.35 a
Musical song ‘Group B monsters—with pure engine sounds’ ~25 cm	Female tunnel lengths	27.00 ± 5.29 a
	Number of progeny per beetle pair	8.20 ± 1.15 a
Musical song ‘Group B monsters—with pure engine sounds’ ~40 cm	Female tunnel lengths	18.00 ± 3.60 a
	Number of progeny per beetle pair	9.20 ± 2.56 a
1 kHz sin wave ~10 cm	Female tunnel lengths	21.20 ± 12.27 a
	Number of progeny per beetle pair	11.26 ± 5.20 a
1 kHz sin wave ~25 cm	Female tunnel lengths	15.00 ± 6.24 a
	Number of progeny per beetle pair	10.00 ± 1.73 a
1 kHz sin wave ~40 cm	Female tunnel lengths	12.20 ± 10.96 a
	Number of progeny per beetle pair	7.40 ± 2.22 a
15 kHz sin wave ~10 cm	Female tunnel lengths	16.40 ± 10.99 a
	Number of progeny per beetle pair	6.94 ± 2.84 a
15 kHz sin wave ~25 cm	Female tunnel lengths	31.00 ± 18.86 a
	Number of progeny per beetle pair	9.80 ± 2.92 a
15 kHz sin wave ~40 cm	Female tunnel lengths	0.00 ± 0.00 a
	Number of progeny per beetle pair	6.15 ± 0.35 a
Control ~10 cm (no vibroacoustic treatment)	Female tunnel lengths	19.20 ± 23.46 a
	Number of progeny per beetle pair	9.58 ± 6.70 a
Control ~25 cm (no vibroacoustic treatment)	Female tunnel lengths	21.60 ± 22.56 a
	Number of progeny per beetle pair	7.90 ± 2.98 a
Control ~40 cm (no vibroacoustic treatment)	Female tunnel lengths	21.00 ± 4.24 a
	Number of progeny per beetle pair	9.00 ± 5.65 a
